# Health-related quality of life of long-term patients receiving opioid agonist therapy: a nested prospective cohort study in Norway

**DOI:** 10.1186/s13011-020-00309-y

**Published:** 2020-09-03

**Authors:** Christer Frode Aas, Jørn Henrik Vold, Svetlana Skurtveit, Aaron G. Lim, Sabine Ruths, Kamrul Islam, Jan Erik Askildsen, Else-Marie Løberg, Lars Thore Fadnes, Kjell Arne Johansson, for the INTRO-HCV Study Group, Christer Frode Aas, Vibeke Bråthen Buljovcic, Fatemeh Chalabianloo, Jan Tore Daltveit, Silvia Eiken Alpers, Lars T. Fadnes, Trude Fondenes Eriksen, Per Gundersen, Velinda Hille, Kristin Holmelid Håberg, Kjell Arne Johansson, Rafael Alexander Leiva, Siv-Elin Leirvåg Carlsen, Martine Lepsøy Bonnier, Lennart Lorås, Else-Marie Løberg, Mette Hegland Nordbotn, Cathrine Nygård, Maria Olsvold, Christian Ohldieck, Lillian Sivertsen, Hugo Torjussen, Jørn Henrik Vold, Jan-Magnus Økland, Tone Lise Eielsen, Nancy Laura Ortega Maldonado, Ewa Joanna Wilk, Ronny Bjørnestad, Ole Jørgen Lygren, Marianne Cook Pierron, Olav Dalgard, Håvard Midgard, Svetlana Skurtveit, Peter Vickerman

**Affiliations:** 1 Bergen Addiction Research group, Department of Addiction Medicine, Haukeland University Hospital, Østre Murallmenningen 7, N-5012 Bergen, Norway; 2 Department of Global Public Health and Primary Care, University of Bergen, Bergen, Norway; 3 Norwegian Centre for Addiction Research, University of Oslo, Oslo, Norway; 4 Department of Mental Disorders, Norwegian Institute of Public Health, Oslo, Norway; 5 Population Health Sciences, Bristol Medical School, University of Bristol, Bristol, UK; 6 Research Unit for General Practice, NORCE Norwegian Research Centre, Bergen, Norway; 7 Department of Social Sciences, NORCE Norwegian Research Centre, Bergen, Norway; 8 Department of Economics, University of Bergen, Bergen, Norway; 9 Department of Clinical Psychology, Medicine, University of Bergen, Bergen, Norway; 10 Division of Psychiatry, Haukeland University Hospital, Bergen, Norway

**Keywords:** Health related quality of life, Quality of life, EQ-5D, Opiate substitution therapy, Opioid agonist therapy, Opioid dependence, Epidemiology

## Abstract

**Background:**

Opioid dependence carries the highest disease burden of all illicit drugs. Opioid agonist therapy (OAT) is an evidence-based medical intervention that reduces morbidity and mortality. There is limited knowledge on the health-related quality of life (HRQoL) of long-term patients in OAT. This study measures HRQoL and self-perceived health of long-term patients on OAT, compares the scores to a Norwegian reference population, and assesses changes in these scores at 1-year follow up.

**Methods:**

We conducted a nested prospective cohort study among nine OAT outpatient clinics in Norway. 609 OAT patients were included, 245 (40%) followed-up one year later. Data on patient characteristics, HRQoL, and self-perceived health was collected. HRQoL was assessed with the EQ-5D-5L, which measures five dimensions (mobility, self-care, usual activities, pain/discomfort and anxiety/depression) on a five-point Likert scale (from “no problems” to “extreme problems”). An UK value set was applied to calculate index values (from 0 to 1) for the EQ-5D-5L and compare them to a Norwegian reference population. Self-perceived health was measured with EQ-VAS (from 0 to 100).

**Results:**

Mean (standard deviation (SD)) EQ-5D-5L index value at baseline was 0.699 (0.250) and EQ-VAS 57 (22) compared to 0.848 (0.200) and 80(19) for the Norwegian reference population. There were large variations in EQ-5D-5L index values, where 43% had > 0.8 and 5% had < 0.2 at baseline. The lowest EQ-5D-5L index values were observed for female patients, age groups older than 40 years and for methadone users. At follow-up, improvements in HRQoL were observed across almost all dimensions and found significant for mobility and pain/discomfort. Mean (SD) overall index value and EQ-VAS at follow up were 0.729 (0.237) and 59 (22) respectively.

**Conclusion:**

The average HRQoL and self-perceived health of OAT patients is significantly lower than that of the general population, and lower than what has been found among other severe somatic and psychiatric conditions. Around 34% had very good HRQoL, higher than average Norwegian values, and around 5% had extremely poor HRQoL.

## Background

Opioid dependence is a severe chronic relapsing disorder consisting of a cluster of physiological, behavioral, and cognitive phenomena [1]. Worldwide, opioid use disorders affect over 16 million people and are responsible for over 120.000 deaths per year [2]. Of all illegal drugs, opioids denote the highest disease burden, have the highest demand for treatment, contribute to substantial increased healthcare costs, and have given rise to a marked increase in opioid related deaths in the last decade [3–5]. People with opioid use disorders suffer not only from a shorter life-span as compared to the general population, but also severe social marginalization and long-term impairments in most aspects of their lives [6]. Research consistently shows that people with opioid use disorders have inferior quality of life (QoL) compared to the general population [7, 8]. This is partly explained by the extensive co-occurrence of substance use disorder and mental disorders, which both seem underdiagnosed and undertreated [9], in addition to high prevalence of somatic disorders such as chronic hepatitis C of almost 50% [10]. Epidemiological studies suggest a prevalence of around 27% for anxiety disorders, 35% for affective disorders, 30% for attention-deficit hyperactivity disorder, and 51% for personality disorders in patients with substance use disorders [11–13]. However, prevalence may be even higher in clinical studies as people with severe problems are more likely to seek help; studies have found prevalence of around 70% for one or more personality disorder [14] and around 66% for childhood trauma among people with substance use disorders [15], and at least one comorbid psychiatric disorder in approximately 80% of patients on opioid agonist therapy (OAT) [16].

Increased focus on, and availability of harm reduction programs, such as OAT have lowered the demand for heroin in Western Europe including Norway [5]. OAT is an evidence-based medical intervention that reduces illicit opioid use, improves patients’ health and reduces crude mortality rates significantly [3, 17–20]. For instance, results of 22 pooled longitudinal cohort studies showed a crude mortality rate for patients on OAT of 0.90 per 100 person years, compared to 1.63 when OAT was ceased and 4.91 for untreated periods [21]. Most research on OAT has emphasized on crude mortality rate, abstinence and retention in treatment, rather than what may be most important for each individual patient; personal wellbeing. In turn, several researchers argued that health related quality of life (HRQoL) should be included as an outcome when evaluating substance use and OAT treatment [22–25]. Thus, to evaluate real life outcome of OAT, changes in objective and self-perceived health, including the individual’s own experience, should be examined. In addition, for more individualized OAT treatment and management, it is important to understand the relationship between clinical and demographic characteristics and HRQoL.

Factors associated with poor HRQoL among OAT patients are older age, female gender, and mental and physical comorbidity [7, 26, 27]. There is building evidence that HRQoL is substantially lower among people with opioid dependence and that HRQoL improves at OAT initiation and during the first few months of treatment [27–30]. However, a recent systematic review suggests there is still limited knowledge regarding HRQoL outcomes in OAT treatment programs and HRQoL outcomes are rarely used [22]. Many of the previous studies are cross-sectional rather than longitudinal designs, offers few participants and with non-validated HRQoL measures for opioid dependence, which make comparisons difficult across opioid dependence and other diseases.

The principal aim of this study is to evaluate the HRQoL and self-perceived health of a large cohort of long-term patients with opioid dependence enrolled in an integrated OAT program in Norway. The HRQoL of OAT patients will also be compared to that of the general population in Norway. Finally, an assessment of changes in HRQoL and self-perceived health at one-year follow-up will be conducted.

## Methods

### Study design and setting

This study is a nested prospective cohort study linked to the Integrated Treatment of Hepatitis C study (INTRO-HCV) [31]. The observational study recruited participants from May 2017 until January 2020 [31]. HRQoL baseline data was collected at the first OAT health assessment, and follow-up data was collected one-year after baseline for each patient. Trained research nurses, who were not responsible for clinical patient follow-up, collected the research data via structured patient interviews. The data was recorded directly in an electronic data entry system (CheckWare). The study took place in Bergen and Stavanger, which are cities in southwestern parts of Norway with around 280,000 and 130,000 inhabitants each. The target population was individuals with opioid dependence who received OAT treatment and care in all together nine OAT outpatient clinics. The clinics have adopted an integrated treatment and care model where patients are charted on a nearly daily basis by health professionals; including social workers, specialized and general nurses, psychologists, and physicians specialized in addiction medicine. OAT medications include mostly methadone or buprenorphine-based medications, often with directly observed intake [32].

### Study sample

The study sample included individuals diagnosed with opioid dependence according to International Classification of Diseases version 10 (ICD-10) [33], currently enrolled in OAT treatment, aged 18 years or older, and have given a written informed consent to participate in the study. Individuals were eligible for inclusion regardless of the type of OAT medication or administration form. Remuneration, of around euro 20, was provided once for the participants upon inclusion to participate in the study. Of the 900 patients invited, a total of 609 (68%) patients completed the EQ-5D-5L questionnaire at baseline, and of those, 245 (40%) were followed up with a follow-up questionnaire approximately 1 year after the first visit. Nineteen patients (2%) were excluded because they did not complete the interview or due to missing data of the EQ-5D-5L instrument The mean time between the first and second annual OAT assessment was 375 days (95% confidence interval (CI): 359–392 days). See Table 1 for details on clinical and demographic characteristics and additional file 1 for flowchart of study sample.

**Table 1:** Baseline characteristics of study sample

	Percentages or Mean (SD)
**Patients, n**^a^	
**Gender,** ***n*** **= 609**	
Male	71%
Female	29%
**Age,** ***n*** **= 609**	
< 25	3%
26–40	39%
41–60	53%
≥ 61	5%
Mean age (SD)	44 (10)
**Current OAT medication,** ***n*** **= 588**	
Methadone	38%
Buprenorphine	56%
Buprenorphine/naloxone	4%
Other	2%
Duration of OAT treatment in years, mean (SD), *n* = 583	7.9 (5.4)
**Background demographics**	
Highest level of completed education, *n* = 588	
Did not complete primary and secondary school	5%
Completed primary and secondary school	45%
High school	40%
Undergraduate education ≤3 years	8%
Postgraduate education ≥3 years	2%
Main source of income, several answers possible, *n* = 611	
Paid work (full time or part time)	7%
Sick pay or unemployment benefits	10%
Social or disability benefits	79%
Savings or scholarships	1%
Other	3%
Accommodation last 30 days several answers possible, *n* = 606	
Owned property	9%
Rented property	68%
Temporary property	7%
Prison	1%
Homeless	1%
At friends or family	12%
Other	2%
Living conditions *n* = 586	
Living alone	63%
Living with others	37%
Children *n* = 587	
Do not have children	44%
Have children	56%
For those having children < 18 years old, *n* = 152	
Having children < 18 with visiting rights	79%
Having children < 18, but no visiting rights	21%

*OAT* Opioid agonist therapy, *SD* Standard deviation,

^a^n = number of respondents, some questions allow multiple answers

### Instruments

#### Health related quality of life: EQ-5D-5L

The EQ-5D-5L instrument is a widely used generic measure of HRQoL [34] and validated for opioid use disorders [35, 36]. It consists of two components. The first descriptive system evaluates health in five dimensions (Mobility, Self-care, Usual activities, Pain/Discomfort, Anxiety/Depression). Each dimension has five levels of response, ranging from no problems, slight problems, moderate problems, severe problems, to extreme problems [37]. The second part of EQ-5D-5L entails a visual analogue scale (VAS) where the respondent rates the self-perceived health from 0 (worst health imaginable) to 100 (best health imaginable) [37]. A systematic review supports the use of the EQ-5D-5L in a broad range of patients [38]. We therefore selected this instrument to assess the HRQoL of patients in OAT and to compare their HRQoL to the general population.

### Statistical analysis

Responses to the five HRQoL dimensions are coded as a five-digit code, which represents a numerical description of a health state. The digits have no arithmetic properties and therefore a single summery number (an index value) needs to be arrived by applying a formula with an appropriate value set, which is a representative sample of the general population. The index value then represents how good or bad a health state is according to the preferences of the general population, ranging from 1 (full health) to 0 (dead, with negative values indicating health states worse than death) [37]. In the absence of a Norwegian value set, we applied an EQ-5D-5L value set for UK, i.e. the societal preference weights for the health state, to determine the EQ-5D-5L index values for each health state in the OAT cohort [39]. Summary statistics were derived, including proportions and number of patients for the five EQ-5D-5L dimensions by age, gender and OAT medications. The EQ-VAS score was summarized descriptively by mean, standard deviation (SD), minimum and maximum as the data was not particularly skewed. A paired t-test of means for the 245 patients with two time points was used in the analysis to investigate whether there was any statistical significance in EQ-5D-5L between the measurements. An ANCOVA model for EQ-VAS changes from baseline to the next OAT health assessment was conducted where place and treatment were fixed effects and baseline covariate. If data were missing from more than one dimension participants were excluded. Altogether eight patients missed data on one dimension at baseline but were included in the analyses. There were no missing data from EQ-5D-5L follow-up or EQ-VAS. To estimate the unbiased treatment effects from baseline to follow-up we used an inverse probability weighted method as we had follow-up data for a subgroup. We calculated population weights based on age, gender and how many times OAT medication was collected during a week in a binominal regression model with follow-u*p* values as the dependent variable. More weight was given to cases with valid data, which were associated with highest probability of having missing data, and less weight was given to cases with lowest probability of missing. The mean for the population weights was 1.0 (SD 0.12) in our model. Statistical significance was set at *p* < 0.05 level. All analyses were made with STATA SE 16.0.

## Results

### Baseline characteristics of study sample

The patients were predominantly male (71%) with a mean (SD) age of 44 years (10). The age range of the study sample was 23–74 years. Most received buprenorphine-based medication (60%) followed by methadone (38%). Duration in OAT treatment ranged from 0 to 25 years, with a mean (SD) of 7.9 (5.4) years. Results therefore reflects HRQoL of patients that have received OAT over a long period of time. Of the participants, 45% had completed secondary school and 40% completed high school. Almost 80% received either disability pension or social benefits as main source of income. Of the 152 OAT patients with children under 18 years, 21% reported they had no visiting rights (Table 1). The distributions of sociodemographic variables were similar for the baseline- and the follow-up samples. However, the proportion of males increased from 71 to 76% in the follow-up sample and mean (SD) age increased from 44 (10) to 45 (10) years compared to the sample at baseline.

### HRQoL of OAT patients at baseline

**Fig. 1:**
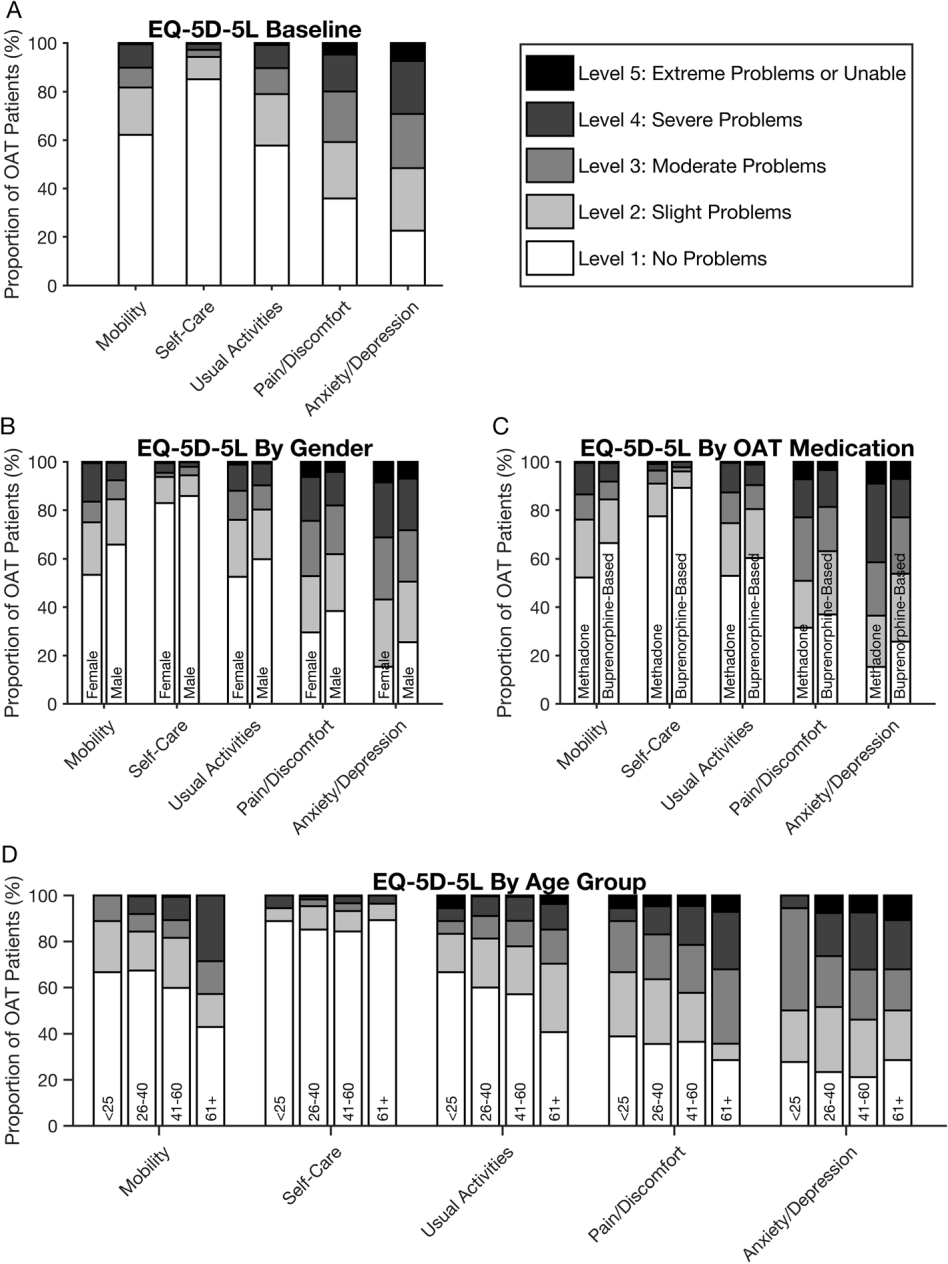
Proportion of individuals reporting problems by EQ-5D-5L domain; overall, by age, gender and OAT medication. OAT = opioid agonist therapy. Altogether 609 respondents, 8 patients missed values on one dimension. 11 patients, which did not receive either methadone or buprenorphine-based medications are left out of the illustration but not analysis. Proportions (%) and dimensions (mobility, self-care, usual activities, pain/discomfort and anxiety/depression). 1A: Overall, 1B: Gender 1C: OAT medication 1D: Age groups

The distributions of unadjusted EQ-5D-5L scores are presented as norm sets according to gender, age groups, and OAT medications (Fig. 1). Overall, mean scores for the five dimensions were 1.7 (95% CI: 1.6–1.8) for mobility, 1.3 (95% CI: 1.2–1.3) for self-care, 1.8 (95% CI: 1.7–1.9) for usual activity, 2.3 (95% CI: 2.2–2.4) for pain/discomfort and 2.7 (95% CI: 2.6–2.8) for anxiety/depression (additional file 2). “No problems” were reported by 62% for mobility, 85% for self-care, 58% for usual activities, 36% for pain/discomfort, and only 23% for anxiety/depression. This means that the majority of patients had no problems with mobility and conducting usual activities and self-care. On the other hand, extreme problems” with pain/discomfort were noted by 5 % and 7 % reported “extreme problems” with anxiety/depression. Under 1 % reported “extreme problems” with mobility, self-care and usual activities. Females and patients receiving methadone treatment reported more problems across all EQ-5D-5L domains compared to males and patients on buprenorphine-based medications, respectively. Patients in the age group 41–60 reported more problems on every domain except pain/discomfort compared to patients under 40 years of age, while patients over 60 years of age reported most problems for mobility and pain/discomfort (additional file 2).

**Fig. 2:**
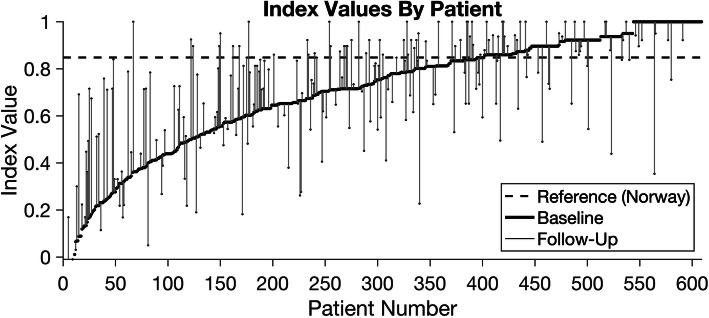
Pen’s Parade: distribution from lowest to greatest health. Pen’s Parade: The distribution in baseline EQ-5D-5L index values, where 43% had > 0.8 and 5% had < 0.2 at baseline. The dotted line represent the average reported index value of the Norwegian reference population

The mean (SD) EQ-5D-5L index value for OAT patients was 0.699 (0.250) at baseline. Forty-three percent had an index value above 0.8, meaning they had “no problems” in the five health domains. Thirty-four percent of the sample even had an index value above 0.848 (Norwegian reference population [40]), meaning their HRQoL was better than that of the Norwegian general population. Around 5 % had an index value below 0.2, meaning they had “extreme problems” in HRQoL. The distribution in baseline EQ-5D-5L index values are shown in the Pen’s Parade (Fig. 2). The parade shows the HRQoL distribution, and is defined as a succession of every OAT patient included, with their height proportional to their EQ-5D-5L index value, from lowest to highest.

The mean (SD) EQ-VAS score of OAT patients was 57 (22) for the total sample at baseline, meaning their self-perceived health was considerably lower compared to the Norwegian reference population of 80 (19) [40]. Females reported an EQ-VAS of 56 (23), while 54 (22) for patients aged 41–60, and 51 (23) for patients older than age 60. Patients on methadone reported 53 (22), which was lower EQ-VAS compared to buprenorphine with 58 (22) and buprenorphine-naloxone 65 (21).

### Changes in HRQoL of OAT patients at follow up

Altogether 245 (40%) of the 609 patients at baseline were included for the follow-up analyses. As shown in the Pen’s Parade (Fig. 2), individual changes in EQ-5D-5L index values for patients with follow-up data (*n* = 245) are indicated with vertical lines. For instance, a patient with an index value of 0.563 at baseline and a long vertical line going up to 0.840 at follow-up means this patient reported a significant improvement in HRQoL. A patient with a vertical line going down from baseline shows worsen HRQoL between baseline and follow-up. Patients with no follow-up data (*n* = 364) or no change at follow-up (*n* = 26) has no vertical line. Figure 2 also shows that the majority of patients have a lower index value than the Norwegian reference population, meaning worse HRQoL, illustrated by values below the dotted line. However, changes go in both directions and appear substantial for some. This means that patients receiving long-term OAT are at risk of relatively rapid changes in index values in both better and worse directions. Overall, around 54% reported improvement in HRQoL, around 35% reported worse HRQoL while 11% reported no changes at follow up compared to baseline values. The mean (SD) observed change was 0.038 (0.20) with minimum and maximum values of − 0.646 and 0.639, respectively. Females reported a mean (SD) change of 0.056 (0.17) compared to males 0.032 (0.21). Variation in individual EQ-5D-5L index value changes from baseline to follow-up is illustrated in additional file 3.

**Fig. 3:**
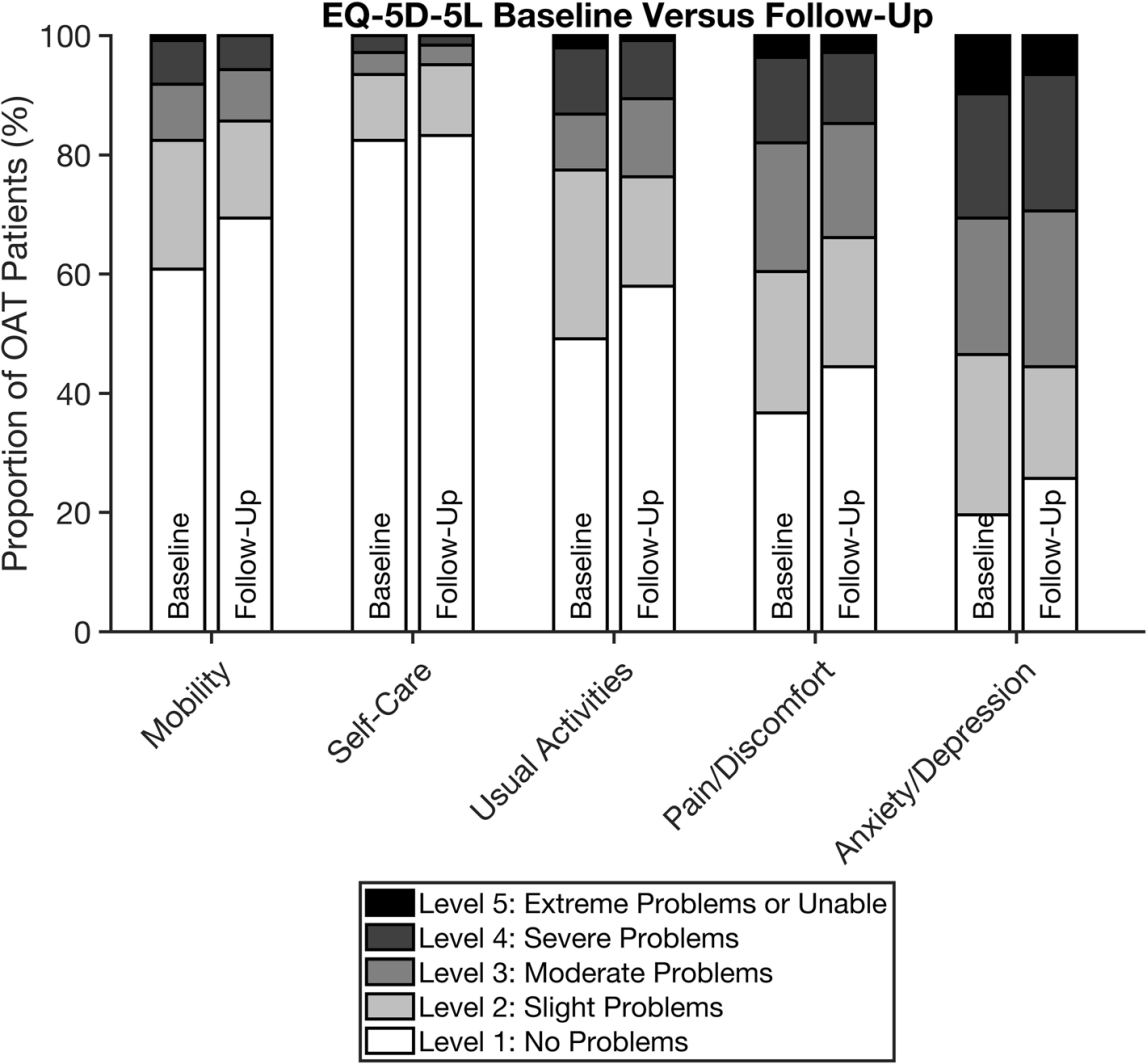
EQ-5D-5L domains at baseline versus follow-up. OAT = opioid agonist therapy. Proportions (%) and dimensions (mobility, self-care, usual activities, pain/discomfort and anxiety/depression). Baseline and follow-up for the 245 patients who were eligible for follow-up analysis

EQ-5D-5L index values improved significantly overall (*p* = 0.004) and for both genders (m: 0.039, f: 0.016), age group 26–40 (*p* = 0.002) and buprenorphine-based patients (*p* = 0.027) as shown in Table 2. The mean (SD) EQ-5D-5L index value was 0.729 (0.237) at follow-up; 49% had an index value above 0.8 while 37% of the sample had an index value above the Norwegian reference population. Around 4 % had an index value below 0.2. Significant improvements in EQ-5D-5L scores were found for mobility (*p* = 0.008) and pain/discomfort (*p* = 0.025) (Fig. 3 and Table 3).

**Table 2:** OAT patients’ HRQoL and self-perceived health at baseline and follow up as measured by the EQ-5D-5L

	EQ-5D-5L VAS, baseline		EQ-5D-5L VAS, baseline^a^		EQ-5D-5L VAS, follow-up		EQ-5D-5L Index		EQ-5D-5L Index		EQ-5D-5L Index	
	n 609		n 245		n 245		n 609, baseline		n 245, baseline^a^		n 245, follow-up	
	Mean	SD	Mean	SD	Mean	SD	Mean	SD	Mean	SD	Mean	SD
**Overall**	57	22	57	22	59	22	0.699	0.250	0.691	0.237	**0.729**	0.237
**Gender**												
Female	56	23	57	25	57	23	0.653	0.260	0.613	0.273	**0.669**	0.261
Male	57	22	57	21	**60**	22	0.718	0.243	0.716	0.220	**0.748**	0.226
**Age group**												
< 25	58	18	55	19	61	25	0.787	0.164	0.766	0.211	0.678	0.390
26–40	61	22	59	22	60	22	0.724	0.242	0.689	0.234	**0.745**	0.205
41–60	54	22	55	22	58	22	0.684	0.253	0.686	0.238	0.716	0.253
≥61	51	23	56	16	58	16	0.613	0.292	0.714	0.257	0.754	0.210
**OAT medication**												
Methadone	53	22	54	21	58	23	0.636	0.260	0.657	0.228	0.686	0.236
Buprenorphine	58	22	58	22	60	22	0.726	0.235	0.716	0.238	**0.758**	0.232
Buprenorphine/ naloxone	65	21	59	30	54	26	0.775	0.242	0.558	0.352	**0.737**	0.335

*OAT* Opioid agonist therapy, *SD* Standard deviation

Index obtained from Devlin, N., Shah, K., Feng, Y., Mulhern, B. and van Hout, B., 2018. Valuing Health-Related Quality of Life: An EQ-5D-5L Value Set for England. Health Economics

^a^Baseline values for the 245 patients who were eligible for follow-up analysis

The possible range of scores for EQ-5D-5L (0–1, 0 = dead (scores < 0 is possible), 1 = full health) and EQ-VAS (0–100, 0 = worst health imaginable, 100 = best health imaginable

Statistically significant changes are marked in bold (*p* < 0.05)

**Table 3:** Distribution of EQ-5D-5L dimensions at baseline and at follow-up

Mean time between baseline and follow-up is 375 days (95% CI: 358,6–391.9)				
Baseline: 609 patients Follow-up: 245 patients				
**Dimension**	**Baseline**	**Baseline**^a^	**Follow-up**	***p*** **value**
**Mobility mean (95% CI)**	**1.7 (1.6–1.8)**	**1.7 (1.5–1.8)**	**1.5 (1.4–1.6)**	
No problems n (%)	378 (62.1)	149 (60.8)	170 (69.4)	0.0081
Slight problems n (%)	119 (19.5)	53 (21.6)	40 (16.3)	
Moderate problems n (%)	49 (8.1)	23 (9.4)	21 (8.6)	
Severe problems n (%)	59 (9.7)	18 (7.4)	14 (5.7)	
Unable to walk about n (%)	3 (0.5)	2 (1.0)	0 (0)	
**Self-care mean mean (95% CI)**	**1.3 (1.2–1.3)**	**1.3 (1.2–1.4)**	**1.2 (1.2–1.3)**	
No problems n (%)	517 (84.9)	202 (82.5)	204 (83.3)	0.4
Slight problems n (%)	56 (9.2)	27 (11.0)	29 (11.8)	
Moderate problems n (%)	18 (2.9)	9 (3.7)	8 (3.3)	
Severe problems n (%)	15 (2.5)	7 (2.9)	4 (1.6)	
Unable to wash or dress n (%)	2 (0.2)	0 (0)	0 (0)	
**Usual activities mean (95% CI)**	**1.8 (1.7–1.9)**	**1.9 (1.8–2.1)**	**1.8 (1.6–1.9)**	
No problems n (%)	350 (57.5)	120 (49.0)	142 (58.0)	0.1
Slight problems n (%)	129 (21.1)	69 (28.2)	45 (18.4)	
Moderate problems n (%)	64 (10.5)	23 (9.4)	32 (13.1)	
Severe problems n (%)	58 (9.5)	27 (11.0)	24 (9.8)	
Unable to do usual activities n (%)	5 (0.8)	5 (2.0)	2 (0.8)	
**Pain/discomfort mean (95% CI)**	**2.3 (2.2–2.4)**	**2.2 (2.1–2.4)**	**2.1 (1.9–2.2)**	
No pain/discomfort n (%)	218 (35.8)	90 (36.7)	109 (44.5)	0.0245
Slight pain/discomfort n (%)	142 (23.3)	58 (23.7)	53 (21.6)	
Moderate pain/discomfort n (%)	127 (20.9)	53 (21.6)	47 (19.2)	
Severe pain/discomfort n (%)	92 (15.1)	35 (14.3)	29 (11.8)	
Extreme pain/discomfort n (%)	29 (4.8)	9 (3.7)	7 (2.9)	
**Anxiety/depression mean (95% CI)**	**2.7 (2.6–2.8)**	**2.7 (2.6–2.9)**	**2.7 (2.5–2.8)**	
Not anxious/depressed n (%)	137 (22.5)	48 (19.6)	63 (25.7)	0.3
Slightly anxious/depressed n (%)	157 (25.8)	66 (26.9)	46 (18.8)	
Moderately anxious/depressed n (%)	136 (22.3)	56 (22.9)	64 (26.1)	
Severely anxious/depressed n (%)	132 (21.7)	51 (20.8)	56 (22.9)	
Extremely anxious/depressed n (%)	45 (7.4)	24 (9.8)	16 (6.5)	

*CI* Confidence interval

*P*-value: based on paired t-test of means for the 245 patients with two time points

^a^Baseline values for the 245 patients who were included for follow-up analysis

Significant improvement in self-perceived health (EQ-VAS) were found for males (*p* = 0.038).

## Discussion

This study is one of the first to examine changes in HRQoL in a sample of long-term OAT patients over a one-year follow-up period. Most studies on HRQoL demonstrate improvements in HRQoL upon treatment entry, but data on long-term patients’ HRQoL is scarce. Considerable impairments in HRQoL and self-perceived health (EQ-VAS) were found in many of the OAT patients. However, large variations in EQ-5D-5L index values were found between individuals, both at baseline and at follow-up. Significant improvement in overall HRQoL was observed at one-year follow-up with around half of the OAT patients reported some improvement in HRQoL while around one-third experienced worse HRQoL at one-year follow up, with great individual variations. Males reported significant improvement in their self-perceived health.

Compared to the general Norwegian population, which reported no problems regarding mobility (85%), self-care (98%), usual activities (82%), pain/discomfort (54%) and anxiety/depression (79%) [40], OAT patients reported in average consistently higher percentages of problems across all EQ-5D-5L domains, especially pain/discomfort and anxiety/depression where only 36 and 23% respectively, reported no problems. The mean (SD) EQ-5D-5L index value for the Norwegian reference population 0.848 (0.200) [40] was considerably higher compared to the OAT patients at baseline. Mean (SD) total EQ-VAS for the OAT patients at baseline was considerably lower than the Norwegian reference population who reported overall mean (SD) of 80 (19); females 80 (20) and older age 77 (19) [40].

Our findings are consistent with prior research, such as Strada *et. al* (2019) study of a large OAT cohort in Germany; found that OAT patients had a lower HRQoL than the general population [41]. Several studies have demonstrated that female OAT patients report worse overall HRQoL compared to males [8, 27]. However it is unclear why that is the case and gender-focused research is urgently needed. Perhaps females are more vulnerable for stigma, traumatizing events or maybe have a poorer function upon entering OAT in the first place. Age is also strongly correlated to poor physical HRQoL [41]. In our sample the mean age was 44, which is consistent with an aging OAT population in Norway [32, 42]. Increased age of OAT patients coupled with poorly reported HRQoL, may place an increased demand for health care services in the future. This raises a debate on how level of OAT and various integrated treatment policies and strategies could better benefit OAT patients. Even if OAT patients treated with methadone reported worse HRQoL than those with buprenorphine, the results should be interpreted carefully. In current Norwegian OAT guidelines buprenorphine is usually recommended as first line substitution medication and considered safer compared to methadone due to its partial antagonistic effect [43]. It is also likely that patients may prefer buprenorphine because it is less sedative than methadone, and that both younger and perhaps more stable patients are dispensed buprenorphine, and as such, results could be highly confounded.

Previous research has revealed that HRQoL improves considerably at OAT treatment initiation and the first few months [44]: however, not much research to date has investigated the long-term effect of OAT on patients’ HRQoL [7, 22, 29]. For instance, one study among patients on methadone maintenance treatment found that QoL increased markedly in the beginning of the observation, but decreased after 6 months [45] while other studies only saw improvements in the beginning of observation [27, 46]. There is therefore the general belief, based on limited data, that once patients are enrolled in OAT, their HRQoL will remain low and does not change substantially anymore. Our study challenges that belief and is among the first to show that changes in HRQoL, including positive changes, are possible. Additionally, our findings also show that many patients had extreme variations in index values from baseline to follow-up, in both positive and negative directions. This suggests that OAT populations are susceptible to severe impairment and also rapid improvements in their HRQoL. Such swift alterations are perhaps less common among other patient groups and future research should examine what causes these changes in HRQoL in long-term OAT patients.

HRQoL in the long-term OAT population was lower in average compared to what is found in other patient groups, such as diabetes type 1 and 2 [47], HIV/AIDS patients [48] and patients with psychiatric comorbidities such as mild to moderate anxiety and depressive disorders, and residual state of bipolar disease [49]. Using the inverse of disability weights (health state valuations) reported in the Global Burden of Disease study (2017), makes comparisons between HRQoL index values and disability weights possible [50]. Research have shown there is a high comorbidity of psychiatric disorders among people with substance use disorders and individuals on OAT [11, 12, 16], while a six-year follow-up study demonstrated that the high psychiatric comorbidity persisted in long-term OAT patients [51]. This may have severe HRQoL impacts and patients with mental disorders may therefore be overrepresented in the lower extreme of the reported index values. Given the wide distribution of severity of disease within the long-term OAT population, treatment needs to be individualized and better adapted to patient functioning and needs. This opts for rethinking and reassessing OAT programs to better facilitate for integrated treatment, which have found to be consistently superior to treatment of substance use and mental disorders with separate treatment plans [52].

Furthermore, as HRQoL profiles of OAT patients are diverse and dynamic this has implications for personalized patient care and the need for regular assessment of HRQoL as an outcome. We need to better understand what drives the extreme and rapid changes in HRQoL in both positive and negative directions among OAT patients. We need to know how to best prevent large drops in index score and how to increase and maintain the increases in index score over time. Additionally, females have worse HRQoL scores compared to men, which indicates that OAT programs should particularly focus on how to improve HRQoL of females and find explanations for why females have lower HRQoL. Similarly, we found that patients older than 40 years have worse HRQoL. This shows that we need to re-examine health care needs of older patients are met and how we can address their needs better. This is particularly important as long-term OAT patients are now aging and we need to plan for aging populations receiving OAT.

A strength of this study is the large sample size of long-term OAT patients at baseline who are receiving the same level of integrated OAT treatment across their respective OAT outpatient clinics in the two cities. However, there are also limitations to our study. Follow-up was conducted on a sub-group of the initial sample. To reduce the selection bias due to the loss-to-follow-up we performed an inverse probability weighted method. In general, it is problematic to compare HRQoL between studies as setting, population, and level of OAT integrated treatment varies and different instruments are being used. This is also the case for comparisons between results based on EQ-5D-3L and EQ-5D-5L, and when different national value sets are being used. Comparative performance across patient groups is driven by differences in the descriptive systems and associated value sets [37] Another weakness is the absence of a Norwegian value set. Both our study and the study of the Norwegian reference population had to use value sets that resembles the Norwegian population, and for this reason the UK value set was chosen. Studies have confirmed that the latter version of EQ-5D significantly increase both reliability and sensitivity and can potentially reduce the possible ceiling effects encountered in the EQ-5D-3L earlier version [53, 54].

## Conclusion

We found considerably lower HRQoL among long-term OAT patients in average compared to the general Norwegian reference population. However, this is a heterogeneous population. Around one-third had very good HRQoL, higher than average Norwegian values. Improvements in HRQoL were found over the one-year follow-up across most EQ-5D-5L dimensions with some uncertainties on why this was seen. More research is urgently needed to identify and understand why females and older patients have worse HRQoL and shows there is a need for more gender-and age-specific treatment in OAT programs. The wide variations in HRQoL support more emphasis on individualized treatment and personalized patient care, and the need for regular assessment of HRQoL in OAT programs. Our study is among the first to show that changes in HRQoL, including positive changes, are possible even several years after initiation of treatment. Future research should examine what causes these changes in HRQoL in long-term OAT patients.

## Supplementary information

**Additional file 1.** OAT = opioid agonist therapy. Flow chart of study sample.

**Additional file 2.** EQ-5D-5L, descriptive health profile at baseline (frequencies and proportions reported by dimensions and level). OAT = opioid agonist therapy, CI = confidence interval. * = total number of respondents. 8 patients who missed data on one dimension were included in the analysis. ** = long acting morphine sulphate and other opioid prescriptions.

**Additional file 3.** Changes in EQ-5D-5L index value per patient from baseline to follow-up. 609 patients included at baseline, 245 patients at follow-up one year later.

## Data Availability

The INTRO-HCV study is ongoing and as such the dataset is not publically available. However, parts of the dataset used for EQ-5D-5L used for this publication may be available in an anonymous and shortened version upon contacting the corresponding author: Christer F. Aas: christer.frode.aas@helse-bergen.no

## Abbreviations

HRQoL: Health related quality of life
INTRO-HCV: Integrated treatment of hepatitis C virus infection
OAT: Opioid agonist therapy
QoL: Quality of life
