# Supplementary figures and images for: Health-related quality of life of long-term patients receiving opioid agonist therapy: a nested prospective cohort study in Norway
Source: Subst Abuse Treat Prev Policy. 2020 Sep 3;15:68. doi: 10.1186/s13011-020-00309-y (PMC7469909; doi:10.1186/s13011-020-00309-y)

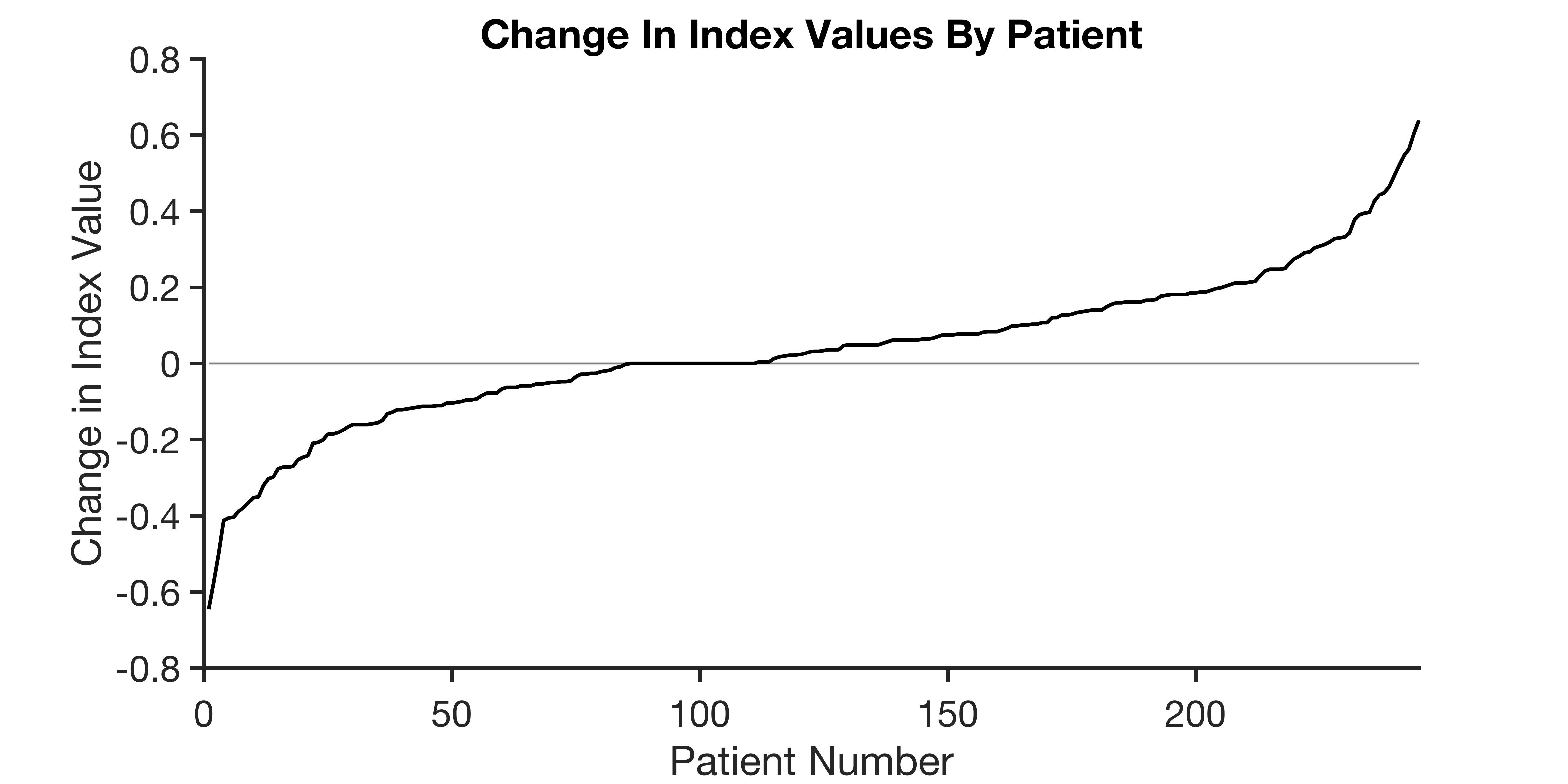

Supplement: Supplementary file 3 — Additional file 3. Changes in EQ-5D-5L index value per patient from baseline to follow-up. 609 patients included at baseline, 245 patients at follow-up one year later. [file 13011_2020_309_MOESM3_ESM.jpg]
